# Supplementary material for: Acute and long-term kidney function after parathyroidectomy for primary hyperparathyroidism
Source: PLoS One. 2020 Dec 31;15(12):e0244162. doi: 10.1371/journal.pone.0244162 (PMC7774859; doi:10.1371/journal.pone.0244162)
Supplement: Attachment — Submitted filename: Rebuttal Letter.docx [file pone.0244162.s002.docx]

# ACUTE AND LONG-TERM KIDNEY FUNCTION AFTER PARATHYROIDECTOMY FOR PRIMARY HYPERPARATHYROIDISM

**REBUTTAL LETTER**

Dear Dr. Itamar Ashkenazi, Academic Editor, 

We appreciate the opportunity to revise and resubmit the manuscript.
We have worked to run multivariate analysis, to short data presentation and discussion, and to review professionally the manuscript.

**5. Review Comments to the Author**

**Reviewer #1:** Well written article regarding a large retrospective cohort of patients operated for PHPT showing in an important percentage various degrees of kidney dysfunction, mostly acutely, but some with long term persistance, suggesting that more cation should be invested in their renal follow-up.

Authors do not mention which was the time span of hyperparathyroidism before surgery.

**Answer: Unfortunately we are unable to clearly state the time span of hyperparathyroidism before surgery. Some patients had unspecific symptoms making difficult to establish for how long they had hyperparathyroidism before their definitive treatment.**

It is also not clear how come so many patients were having MEN1, because he ratio is way above literature data. They should comment on that.

**Answer: University of Sao Paulo is major training center at the country. It is a very specialized facility and it has a long tradition treating hyperparathyroidism related multiple endocrine neoplasia. (reference: Montenegro FLM, Brescia MDG, Lourenço DM Jr, Arap SS, d'Alessandro AF, de Britto E Silva Filho G, Toledo SPA. Could the Less-Than Subtotal Parathyroidectomy Be an Option for Treating Young Patients With Multiple Endocrine Neoplasia Type 1-Related Hyperparathyroidism? Front Endocrinol (Lausanne). 2019 Mar 7;10:123. doi: 10.3389/fendo.2019.00123. PMID: 30899245; PMCID: PMC6417394.)**

Table 1 – authors should underline significant changes compared to baseline at different time points (e.g. p values)

**Answer: We thank for the suggestion**

Table 2 – the information regarding p values needs to be simplified, e.g. * compared to AKI stage 1, # compared to AKI stage 2, $ compared to AKI stage 3. One sign – p < 0.05, two signs – p < 0.01, three signs – p < 0.001.
**Answer: We thank for the suggestion**

Patients with AKI type 3 had clearly higher PTH levels, but also lower 25OH-D3, authors should comment more in detail on that (they already commented in discussion)

**Answer: AKI type 3 patients had more severe HPTP, worse preoperative kidney function and low 25OH-D3 than other groups. In multivariate analysis both preoperative creatinine and 25OH-D3 had influence on acute eGFR variation.**

Authors should make discussions more concentrated and more focused on the main message - that parathyroidectomized patients should be more attentively followed regarding kidney function. They should add separate comments on study limits - retrospective study, small control group, etc. - and conclusions - follow up of kidney function in this type of patients, the effect of surgery itself with decrease of PTH on kidney function, which may be important also in secondary hyperparathyroidism, the importance to revisit vitamin D repletion before surgery in PHPT.

**Answer: We thank the  Reviewer for the suggestions. We shortened discussion and tried to make the relevant points more clear, as well as highlighted study limits.**

Finally, English needs to be improved

**Answer: The manuscript was now professionally revised by the American Journal Experts**

**Reviewer #2:** The aim of this retrospective study, was to investigate both the acute and chronic effects of parathyroidectomy on renal function and relate these to serum biochemistry before surgery in 494 patients with surgically proven PHPT, and to compare with control group.
The authors found that there is a significant acute impairment in renal function after parathyroidectomy for PHPT and almost half of patients meet the criteria for AKI. Significant eGFR recovery was observed during first month after surgery, but a small permanent reduction may occur.

Comments
1. The discussion is too long. Please condense it.

**Answer: We thank for the suggestion and we edited the discussion**

2. Please show serial changes in body weight over time.

**Answer: Unfortunately, due its retrospective method we have no data on body weight over time.**

3. You mentioned that permanent reduction in eGFR occurred in 60.7 % of the patients, after acute episode. Please reveal independent factors contributing to permanent reduction in eGFR by multivariate analysis.

**Answer: We conducted a multivariate model. It is included in the results in order to identify independent influencing factors on acute and long term renal function.**

4. Did you look at blood urea nitrogen (BUN) or uric acid (UA) and their serial changes?

**Answer: No, the regular follow up of these patients did not include BUN or UA tests. The present study is retrospective and we have no such data.**

5. Table 6 showed a significant difference in preoperative GFR between PHPT and control. This is a major limitation. Please describe this as a limitation of this study in the main text.

**Answer: Considering the comment of the reviewer #3 and your comment, we decided to suppress the control group . We added a comment in the discussion that in a previous study we showed that patients undergoing other head and neck procedures had no such change of kidney function. (reference Montenegro FM, Brandao LG, Ferreira GF, Lourenco DM, Jr., Martin RM, Cunha-Neto MB, et al. Transitory increase in creatinine levels after parathyroidectomy: evidence of another action of the parathyroid glands? Arq Bras Endocrinol Metabol. 2011;55(4):249-55. PMID: 217796260)**

6. How do you feel about an association of acute kidney impairment after surgery with renal tubular damage? Or did you look at biomarkers of renal tubular damage, such as beta2 microglobulin or liver-type fatty acid protein?

**Answer: We thank your suggestions of using more specific biomarkers. We will certainly include them in prospective evaluation. These markers are not currently routine in the postoperative care and we don´t have data on them.**

**Reviewer #3**: I commend Belli and col for this interesting article which evaluates changes in renal function following parathyroidectomy for PHPT. The conclusion is interesting and may have important practical consequences. One major strengths of the article is the long in hospital stay and ability to evaluate renal function several days after PHPT. As in most centers PHPT patients are currently operated on an outpatient basis the present study design would be difficult to reproduce.
I have several comments.

1. The authors have included PHPT for adenoma, hyperplasia, MEN1 and parathyroid cancer. Although all these patients have primary hyperparathyroidism these are different diseases with different mechanisms of PHPT and different treatments...I think the article should focus only on patients with PHPT related to parathyroid adenoma which is the most usual cause of PHPT

**Answer: You are quite right MEN1 patients are rather different from  sporadic cases or even the rare parathyroid carcinoma. The extent of the operation is different, being subtotal or total parathyroidectomy with autograft the most common operation in MEN1 patients in contrast to single gland excision in parathyroid adenoma. The operative time is usually longer in multiglandular disease. In addition, MEN1 are usually young age, may have neoplastic comorbidities rather than hypertension (more common in older individuals) and they have a better preoperative renal function. In spite of these facts, we noticed that they also present a mean decrease of their kidney function after parathyroidectomy. We believe this is a very interesting finding, although we are unable to  know the reason. Thus we decide to keep the analysis of these patients.**

2. I Don't see the utility of the control group. Thyroidectomy can result in ablation of one parathyroid gland without significant impact on the overall parathyroid function.

**Answer: We agree with your comment and considering the limitation we excluded this control group. We had a previous retrospective study that has already shown other control groups.**

3. The result section is much too long. as a result it is quite difficult to read.I think that the chapters on relationships between eGFR and AKI, CKD and vit D should only be cited and presented in Tables. Tables and Figures should be counted separately and some should be published as supplementary material because there are too many of them..

**Answer: We shortened data presentation and discussion.**

4. I think an uni/multivariate analysis would be helpful in identifying patients at risk of developing renal disfunction after parathyroidectomy for PHPT.

**Answer: According to your suggestion we included a multivariate analysis. Indeed we found some factors related to acute change and also to long-term changes.**

5. How many patients developed clinically relevant renal failure? How were they managed? Could the authors identify risk factors for such complication?

**Answer: Some patients with preoperative reduced renal function had more clinically concerning acute changes. One patient required dialysis after parathyroidectomy (0.2%) The group has previous case reports published with two patients of 183 (1.1%) operated on from 1997 to 2007 requiring acute dialysis after the procedure. We believe that the difference from the previous 1.1% to current 0.2% is the improvement in the diagnosis of primary HPT leading to an increase in the number of operations with less severe disease. Indeed, as we wrote in the manuscript, in the old days of hyperparathyroidism, Fuller Albright reported marked oliguria in some patients after parathyroidecotmy. (details are available in Montenegro F, Martin R, Lourenço DM Jr. The parathyroid and the kidney: Fuller Albright revisited. World J Surg. 2009 Feb;33(2):246-7. doi: 10.1007/s00268-008-9824-1. PMID: 19020930.)**

6. The discussion is much too long. the authors should stick to their results rather than discuss tertiary parathyroidism and chronic kidney disease....2 or three pages are enough in my opinion

**Answer: We shortened discussion.**

We hope we have been able to respond to the questions and are looking forward to a positive response.

I reiterate that I am available for any doubts regarding the manuscript and make available the raw data if desired by editorial office.

Thank you.
Best regards,

Marcelo Belli, M.D., ph.D.
